# Therapeutic Subthalamic Nucleus Deep Brain Stimulation Reverses Cortico-Thalamic Coupling during Voluntary Movements in Parkinson's Disease

**DOI:** 10.1371/journal.pone.0050270

**Published:** 2012-12-26

**Authors:** Josh Kahan, Laura Mancini, Maren Urner, Karl Friston, Marwan Hariz, Etienne Holl, Mark White, Diane Ruge, Marjan Jahanshahi, Tessel Boertien, Tarek Yousry, John S. Thornton, Patricia Limousin, Ludvic Zrinzo, Tom Foltynie

**Affiliations:** 1 Sobell Department of Motor Neuroscience and Movement Disorders, UCL Institute of Neurology, London, United Kingdom; 2 Lysholm Department of Neuroradiology, National Hospital for Neurology and Neurosurgery UCLH NHS Foundation Trust, London, United Kingdom; 3 Neuroradiological Academic Unit – Department of Brain Repair and Rehabilitation, UCL Institute of Neurology, London, United Kingdom; 4 Victor Horsley Department of Neurosurgery, National Hospital for Neurology and Neurosurgery UCLH NHS Foundation Trust, London, United Kingdom; 5 Wellcome Trust Centre for Neuroimaging, UCL Institute of Neurology, London, United Kingdom; 6 UCL Institute of Cognitive Neuroscience, University College London, London, United Kingdom; 7 Department of Neurosurgery, Medical University of Graz, Graz, Austria; University of Cambridge, United Kingdom

## Abstract

Deep brain stimulation of the subthalamic nucleus (STN DBS) has become an accepted treatment for patients experiencing the motor complications of Parkinson's disease (PD). While its successes are becoming increasingly apparent, the mechanisms underlying its action remain unclear. Multiple studies using radiotracer-based imaging have investigated DBS-induced regional changes in neural activity. However, little is known about the effect of DBS on connectivity within neural networks; in other words, whether DBS impacts upon functional integration of specialized regions of cortex. In this work, we report the first findings of fMRI in 10 subjects with PD and fully implanted DBS hardware receiving efficacious stimulation. Despite the technical demands associated with the safe acquisition of fMRI data from patients with implanted hardware, robust activation changes were identified in the insula cortex and thalamus in response to therapeutic STN DBS. We then quantified the neuromodulatory effects of DBS and compared sixteen dynamic causal models of effective connectivity between the two identified nodes. Using Bayesian model comparison, we found unequivocal evidence for the modulation of extrinsic (between region), i.e. cortico-thalamic and thalamo-cortical connections. Using Bayesian model parameter averaging we found that during voluntary movements, DBS reversed the effective connectivity between regions of the cortex and thalamus. This casts the therapeutic effects of DBS in a fundamentally new light, emphasising a role in changing distributed cortico-subcortical interactions. We conclude that STN DBS does impact upon the effective connectivity between the cortex and thalamus by changing their sensitivities to extrinsic afferents. Furthermore, we confirm that fMRI is both feasible and is tolerated well by these patients provided strict safety measures are adhered to.

## Introduction

Deep brain stimulation of the subthalamic nucleus (STN DBS) is now a recognized treatment for patients experiencing the motor complications of Parkinson's disease (PD) [1]–[3]. However, its mechanisms of action remain unclear. High frequency stimulation has been found to both inhibit and excite different neurons within the target nucleus, having different effects on different neural elements [4]. Evidence exists for both orthodromic stimulation of STN efferents, as well as for antidromic stimulation of STN afferents [5], [6]. In addition, abnormal *patterns* of synchronized firing in the STN observed in PD patients are suppressed by STN DBS [7], [8]. Its powerful neuromodulatory effect is likely due to a combination of these phenomena.

However, its modulatory effect is not limited to subcortical structures; neuroimaging studies have revealed that STN DBS induces widespread changes across the brain. Radiotracer-based imaging methods (positron emission tomography, PET, and single photon emission computed tomography, SPECT) have identified regional changes in blood flow and glucose uptake, believed to be indicative of altered neural demands secondary to a change in activity. Regarding movement-related activity, (i.e. neural activity related to the performance of a motor task), PET studies have demonstrated that DBS increases activity in the rostral supplementary motor area (SMA), anterior cingulate cortex (ACC) and dorsolateral prefrontal cortex (DLPFC) [9]–[12]. Additional changes have been noted in the cerebellum [13], [14], as well as within the subcortical structures composing the basal ganglia [11], [15] (*critically reviewed in* [16], [17]). Reports have varied regarding these modulated areas; this is likely due to different motor tasks, imaging modalities, sample sizes, and significance thresholds used across studies. Additionally, despite the use of stereotactic guidance, DBS is dependent on the precise targeting of subregions of within deep brain nuclei. Different surgical teams adopt slightly different approaches (e.g. micro-electrode recording vs. image-guided techniques [18]), that may influence targeting accuracy and sub-regions within the target that are stimulated. Given the relationship between structure and function, this will inevitably lead to slightly different neural response profiles.

While altered regional responses have been explored, relatively little is known about the effect of DBS on the connectivity within functionally specialised networks. Neural processing is dependent upon functional integration, that is, finely tuned collaboration between functionally specialized regions [19]. Increased *functional* connectivity (i.e. a statistical dependency between regions) in frontal-temporal-parietal-striatal-thalamic networks has been reported in response to DBS of the fornix in patients with Alzheimer's disease, and of the external pallidum (GPe) in patients with Huntington's disease [20], [21]. Changes in the *effective* connectivity between brain regions (i.e. the directed influence one region has over another region's activity) has yet to be explored, and could be more important for understanding the effects of DBS than the regionally specific changes in movement-related responses that they induce.

Functional MRI has advantages over tracer-based imaging including a superior spatial resolution as well as valuable data modeling methods. Its use in these patients however has previously been limited by safety concerns. Interactions between MRI scanners and DBS hardware may induce movement, heating, and electrical currents within the implanted conductors. This could potentially result in severe neurological disability, as well as confound neurostimulator function [22]. As a result, only a handful of DBS patients have been evaluated using fMRI, all during the peri-operative period, without internalized neuro-pacemakers (IPGs), that is, before therapeutic stimulation had been established. However our own *on-site* studies have now confirmed that fMRI can be safely performed during active DBS with a completely internalized system, *provided strict procedures are followed* [23].

We therefore set out to confirm the technical feasibility of fMRI during therapeutic deep brain stimulation (DBS) of the subthalamic nucleus (STN) in patients with Parkinson's disease with a view to then identify if and how STN DBS modulates effective connectivity between regions of altered brain activation during voluntary movements. Specifically, we explored whether any change in effective connectivity resulted from modifying the sensitivity of cortical and thalamic regions to their extrinsic afferents, and/or from modulating these regions' intrinsic sensitivity. We found explicit evidence for the modulation of cortico-thalamic and thalamo-cortical connections, as well as these regions' intrinsic connectivity during voluntary movements in these patients, confirming for the first time that DBS does impact upon cortico-subcortical effective connectivity.

## Materials and Methods

### Ethics statement

This study was approved by the National Hospital and Institute of Neurology Joint Ethics committee (approval number 09/H0716/51). All participants provided written informed consent.

### Patients

Ten PD patients took part in this study (*Table 1*). All patients had PD meeting UK brain bank criteria, and had received bilateral STN DBS for at least 6 months. Surgery had been performed using stereotactic MRI for both preoperative targeting and immediate postoperative verification of lead location prior to implantation of the extension cables and the implanted pulse generator (IPG) [1], [24].

**Table 1:** Clinical Response to STN DBS: Details of 10 PD participants and their motor response to STN DBS, assessed immediately before the fMRI scan while off medication.

Subject	Age	Months since surgery	Dominant Hand	Clinical UPDRS-III Off/OFF	Clinical UPDRS-III Off/ON	UPDRS % Improvement	Movement Durations/s			Reaction Time/s		
							OFF Stim	ON Stim	% Change	OFF Stim	ON Stim	% Change
1	65	20	Left	53	21	60.4	1.17	1.32	12.82	0.77	0.98	27.27
2	72	53	Right	47	29	38.3	2.38	0.70	−70.59	1.00	0.56	−44.00
3	54	9	Right	33	10	69.7	0.80	0.59	−26.25	0.56	0.49	−12.50
4	65	67	Right	60	20	66.7	0.98	0.69	−29.59	0.55	0.42	−23.64
5	50	102	Left	51	17	66.7	1.26	0.83	−34.13	0.62	0.49	−20.97
6	63	29	Right	46	19	58.7	0.62	0.50	−19.35	1.10	0.61	−44.55
7	54	19	Right	45	26	42.2	1.00	0.83	−17.00	0.79	0.79	0.00
8	56	30	Left	52	19	63.5	2.39	1.23	−48.54	1.27	0.61	−51.97
9	43	48	Left	51	23	54.9	0.94	0.64	−31.91	0.66	0.62	−6.06
10	61	8	Right	46	25	45.6	1.12	0.85	−24.11	0.93	0.68	−26.88
***Mean***	*58.3*	*38.5*	*N/A*	*48.4*	*20.9*	*56.7*	*1.27*	*0.82*	*−28.86*	*0.83*	*0.63*	*−20.33*
***SD***	*8.51*	*29.5*	*N/A*	*7.03*	*5.32*	*11.1*	*0.62*	*0.27*	*21.51*	*0.24*	*0.16*	*23.91*

‘Off/OFF’ = Off medication, OFF stimulation’; ‘Off/ON’ = Off medication, ON stimulation.

### Stimulation equipment

All patients had bilateral STN electrodes (model 3389, Medtronic, Minneapolis) and a dual channel IPG (*Kinetra™* or *ActivaPC™*, Medtronic, Minneapolis) implanted. Stimulation parameters had been previously optimised according to clinical response. Inclusion in this study was restricted to those patients who (1) could tolerate lying flat while being *both* off medication and off stimulation, (2) exhibited minimal head tremor, and (3) demonstrated an immediate >35% improvement in UPDRS part 3 (UPDRS-III) off-medication score when stimulation was switched ON compared with OFF. Medication was withdrawn for 10–12 hours (overnight) before the scanning session.

Before scanning, (1) UPDRS-III motor scores were documented both ON and OFF stimulation (OFF was scored approximately 10 minutes after stimulation was stopped), (2) stimulation parameters and system impedance were recorded, and (3) IPG counters were reset.

Participants wore MRI compatible isolating headphones and held an MRI compatible joystick in one hand (Cambridge Research Systems, Kent, England: model No: HH-JOY-4. Angular range: 30 degrees (+/−15 degrees), Grip: 11.5×3 cm). The position of the joystick in time and space was recorded at a sampling rate of 20 Hz. During the task, participants were instructed to move the joystick consistently in response to auditory signals and to avoid excessively fast or large movements. Their heads were securely supported using a vacuum moulded cushion to dampen any head movement. Patients held an alarm in their non-moving hand to alert the clinical team if they experienced any discomfort during the scan. Patients were asked to keep their eyes closed throughout scanning.

The task was performed both with therapeutic stimulation active (ON), and again when their stimulation inactivated (OFF). During each stimulation condition, the task was performed twice, once with each hand. In other words, every patient performed a right and left hand movement task while stimulation was ON and OFF. The order of stimulation (ON versus OFF) and the movement (right versus left) were randomised over subjects. DBS was switched ON or OFF using the patients' own *Access™* controller, which we ensured functioned normally within the MRI environment.

### MRI data acquisition

All scans were performed with a Siemens Avanto 1.5T MRI scanner (Siemens, Erlangen, Germany) using a Siemens-supplied transmit-receive (T/R) head coil, similar to the one that detailed tissue-equivalent test-object thermometry experiments had been performed with in our previous safety study [23]. The specific absorption ratio (SAR) in the head was limited to under 0.1 W/Kg.

Each participant completed the session with four movement task time-series (sessions), one for each hand during each stimulation condition. This corresponds to a factorial design with three factors; task (movement versus no movement); laterality (right versus left) and stimulation (ON versus OFF). The whole session took approximately 90 minutes. The connection between the electrode lead and the extension cable, commonly sited above the left parietal bone caused a loss of signal artefact resulting in data not being acquired in left hemispheric sensorimotor areas. Given these regions were *a priori* regions of interest, particularly when examining right hand movements, we elected only to analyse the left hand movement data. Acquisition parameters were as follows:

1. T1 weighted magnetization-prepared rapid gradient-echo (MPRAGE) structural scan (repetition time TR = 1590 ms, echo time TE = 3.3 ms, inversion time TI = 1100 ms, flip angle = 15°, field of view FOV = 250×250 mm^2^, matrix size = 192×192, 144 sagittal slices 1.3 mm thick, for a spatial resolution of 1.3 mm isotropic) lasting approximately 10 minutes. This scan, and an additional 8 minute resting scan (to be reported elsewhere), allowed a constant period of equilibrium to follow each patient's stimulation adjustment.
2. GE-EPI Movement session, Hand 1: (TR = 3695 ms, TE = 40 ms, flip angle = 90°, FOV = 192×192 mm^2^, matrix size = 64×64, 49 axial slices 2.5 mm thick, gap between slices of 0.5 mm, for a spatial resolution of 3×3×3 mm^3^, 96 volumes, acquisition time = 6 minutes). The fMRI task paradigm consisted of 12 blocks lasting ∼30 seconds each. During each block, a series of 15 audio stimuli (beeps) were sounded through the headphones. The time between beeps was randomised to between 1–3 seconds. The blocks alternated between a “rest” and a “go”. At the beginning of each “rest” block the participant heard the word “rest” and was instructed to rest their hand on the joystick, ignore the beeps and keep still. At the beginning of each “go” block the participant heard the word “go” and was instructed to move the joystick in one of four random directions of their choice, and then return the manipulandum back to the central resting position. A single movement was defined as moving the joystick from its position of equilibrium and then returning the joystick back to this position. The exact timings of the beeps were also recorded.
3. GE-EPI Movement session, Hand 2: The joystick was then moved to the opposite hand, and acquisition 2 was repeated.

Additionally, field maps were acquired to correct for field inhomogeneity. Patients then had their stimulation switched to the opposite condition. The joystick was returned to the hand that had first performed the task and the aforementioned acquisitions were repeated.

At the end of the session, DBS was switched back ON if OFF during the second session, and the patient was examined (including a repeat evaluation of UPDRS-III). The DBS system was interrogated to check the settings and impedance, and to check for additional activations. The patients were given their regular PD medication and had a final clinical assessment after their medication had started to take effect to confirm they had returned to their baseline level of Parkinsonian disability before leaving the department.

Movement durations and reaction times were extracted from the joystick dataset. Paired *t* tests were used to judge significant changes in joystick movements comparing ON and OFF stimulation periods.

### Image processing and regional BOLD signal analysis

Data was pre-processed and analysed using the SPM8 (Wellcome Trust Centre for Neuroimaging, London, UK; http://www.fil.ion.ucl.ac.uk); for effective connectivity analyses, DCM12 was used. The SPM Anatomy toolbox [25] was used to translate *peak* MNI coordinates into anatomical and functional regions based on probabilistic cytoarchitectonic maps.

Data were first corrected using the acquired field maps; accounting for field inhomogeneity caused both by the skull's air sinuses, and the implanted DBS equipment. When we examined the field maps, we noted that the extent and amplitude of the expected distortions caused by the DBS hardware on the skull are approximately the same as the extent and amplitude of the distortions caused by the presence of the sinuses. This suggests that the DBS hardware causes no more distortion than the sinuses do, and the field maps are sufficient to correct for them. Data were then realigned, correcting for motion. Each subject's T1 image was then normalised to MNI space. The normalisation matrix was then used to normalise the functional GE-EPI data. Each of the images was then visually inspected to confirm they had been correctly normalised. This order of normalisation ensured that the functional and anatomical scans were correctly translated into MNI space. The data were smoothed using an 8 mm Gaussian kernel, accounting for variation across subjects in structural and functional anatomy. Low frequency fluctuations were modelled using a high-pass filter set to the standard threshold (128-s).

Standard SPM (whole brain) statistical analyses were then performed using an epoch-related design, where each activation epoch (block) was defined as the time period from the beginning of the first movement in a “go” block, to the end of the last movement in that block. Each movement session thus consisted of six motor epochs, corresponding to the six “go” blocks. The resulting boxcar task function was then convolved with a canonical haemodynamic response function to form expansionary variables or regressors that constitute the design matrix. Both (ON and OFF) movement sessions, for each participant, were analysed in one design matrix. Six nuisance regressors were included for each session modelling the confounding effects of head motion in the design matrix.

We performed a standard random effects analysis by first computing contrasts of effects at the “first level” (within subject) and then analysing these summary statistics at the “second level” (between subjects) using one sample t-tests. Intrinsic masking was used to exclude voxels affected by DBS hardware-related artefact.

We examined for the contrast corresponding to the main effect of movement to (1) ensure that this could be detected in the DBS setup with a suitable degree of sensitivity and anatomical precision, and (2) to define a network of brain regions engaged by the motor task. We then explored the interaction between task and stimulation. This resulted in two contrasts (*Main Effect of Movement* – Left hand, *Movement×Stimulation interaction* – Left hand), and ensuing statistical parametric maps (SPMs).

All 10 subjects' normalised structural T1 scans (taken during ON) were combined to create a group structural T1 normalised to MNI space. One-sample t-tests were performed on group data separately for each of the contrasts to produce SPMs that were then superimposed on the group structural image. Second level tests on the main effect of movement contrast were adjusted for handedness and UPDRS-III ON score by including mean-centred cofounds in the second level design matrix (multiple linear regression model). This accounts for confounding effects due to inter-subject variability in Parkinsonian disability. In the same manner, second level tests on interaction contrasts were corrected for handedness and percentage improvement in UPDRS-III score when going from OFF to ON.

The main effect of movement contrast served to define the network of brain regions related to voluntary movement. The fifteen peak voxels of clusters larger than 5 voxels with the highest z statistics (range: 4.87–3.81) were defined as ‘nodes’ of the motor network. Any clusters consisting of purely white matter voxels were omitted.

A restricted volume analysis was then performed to assess the interaction between movement and stimulation within the nodes of the aforementioned network. Restricted volumes (8 mm radius spheres) were centred on the peak voxel of each node. A statistical threshold of p<0.05 (FWE corrected, with a cluster threshold of 5 voxels) was used to assess significance. Regions surviving this threshold were considered to show robust interactions between movement and stimulation.

Given that extensive PET imaging studies have previously proposed a network of areas involved in the therapeutic response to STN DBS and that this is the first report of fMRI activations and their modulation by therapeutic DBS, we also performed a whole-brain statistical search. From this analysis, regions that survived a criterion of p<0.0005 (uncorrected, cluster threshold of 5 voxels) are reported as discovered areas of interest that will be examined in future studies.

### Effective connectivity analysis

Dynamic causal modelling (DCM) is a Bayesian framework that aims to explain how observed BOLD responses are generated by estimating the effective connectivity between specified regions of interest [26], [27]. DCM models hidden neuronal dynamics using an explicit forward model based on the balloon model [26], [28]. In brief, realistic models of the functional architecture are constructed, the BOLD signal from these regions is extracted and the neuronal (hidden) states are inferred. The effective connectivity between the regions is then expressed in the form of differential equations using three parameter sets; “average connection” parameters (values of the DCM A-matrix), represent latent or average coupling strengths in the absence of experimental manipulation (in our case, average connectivity represents the coupling during voluntary movement), “modulatory” or “bilinear” parameters (values of the DCM B-matrix) denote changes to the average connectivity associated with experimental manipulations (i.e. the additive effect of DBS on coupling strength), and thirdly, “input” parameters (values of the DCM C-matrix) control the effect of driving stimuli by external perturbations (in our case, movements). These parameters are then estimated using Bayesian estimators and are given in Hertz [29]. The coupling parameters represent changes in the sensitivity of one region to afferents from other regions, conceptually comparable to electronic gain; i.e. how much its output changes in response to a given input. DCM has become the method of choice for modelling effective connectivity in neuroimaging data and has been used widely across the literature [30]–[34]. Thus we modelled the effective connectivity between nodes of the motor network that demonstrated regional movement-related increases in BOLD signal that were sensitive to DBS.

The design matrix was finessed (rotated) for the DCM analysis. The left-hand movement ON and OFF scans were concatenated into a single (movement) regressor. Parametric modulators were used to model the movement×stimulation interaction. The main effect of DBS was modelled as a boxcar, with values of one during stimulation ON and zero otherwise.

Subject-specific peak coordinates of the regional interactions were used to identify nodes or regions in the DCM. The inclusion criteria for the DCM analysis required each subject to show a non-trivial interaction in both nodes (n = 7); within subject peaks (p<0.05, uncorrected) were within 16 mm of the second-level (between subject) peaks. Regional activity was summarised as the principal eigenvariate – adjusted for slow fluctuations and other nuisance variables – based on voxels within 4 mm of the subject-specific peaks.

**Figure 1:**
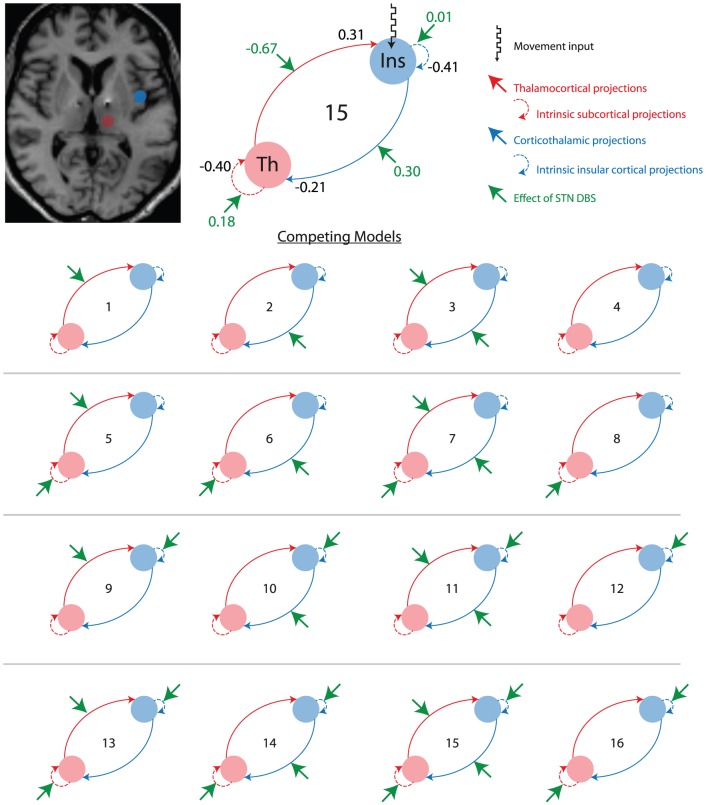
The dynamic causal models compared using Bayesian model selection (model space). Model 15 – the winning model – is shown enlarged. The blue node represents the right insula cortex, and red node, the right thalamus. Green arrows indicate the connection/s that DBS modulates. The ‘movement input’ is likely made up of both motor inputs arriving from M1, PM and SMA, as well as sensory inputs elicited by on-going movements. Thalamic ‘intrinsic subcortical projections’ refer to thalamic afferents from BG nuclei. Cortical ‘intrinsic insula cortical projections’ refer to cortical afferents from within the cortex. Average DCM parameters are included on the enlarged model 15, units are in Hertz (Hz). Positive A-matrix parameters represent an excitatory effect on the target, whereas negative values indicate an inhibition of the target area. Positive B-matrix parameters (value in green) represent an increased target response to input (i.e. an increased gain), whereas negative values indicate a decreased target response to input (i.e. a reduced gain). The coupling during movements with DBS is equal the sum of the A and B value on that connection, e.g. during movements with DBS, the cortico-thalamic drive switches from −0.21 Hz to (−0.21+0.30) = 0.09 Hz, i.e. it switches from an inhibitory to an excitatory drive.

*Figure 1* summarises the different dynamic causal models we evaluated with Bayesian model comparison. Our movement effect entered all models as a driving input to the cortical node. All the areas had intrinsic (within region) and reciprocal extrinsic (between region) connectivity. The modulatory input was set to modulate a subset of connections in each model. These were thus; the forward and backward extrinsic connections between the two regions (identified in the prior restricted volume analysis at FWE corrected p<0.05), and the intrinsic self-connectivity within each region. This resulted in (2^4^ = ) 16 different DCMs per subject, and thus (16 DCMs×7 subjects  = ) 112 DCMs in total. Models were inverted and scored – in terms of their model evidence – using ‘DCM12’.

Bayesian model selection (BMS, fixed-effects assumptions) was then employed to select which of the 16 models had the greatest evidence, given the data collected [27], [35]. BMS computes a Bayes factor for each of the models, allowing us to make inferences about which of several biologically plausible models is optimal given the data [35]. Fixed-effects model comparison was chosen because we selected our subjects under the assumption they have the same functional architecture and that DBS had consistent effects within this anatomy. The free-energy of each model (F) corresponds to the log of the model evidence and indicates the accuracy of the model corrected for its complexity. To assess for the effects of outliers on the BMS, we plotted the relative F values for each model for each subject. Relative values were generated by subtracting the F value of the model with the least evidence, from each model in each subject. In addition, we performed a random-effects BMS for verification to allow for the possibility that different subjects had different connectivity architectures. For quantitative interpretation, the coupling parameters of the DCMs were averaged using Bayesian Model Averaging (BMA), in which parameter estimates are weighted by the model evidence [27].

Finally, we examined the relationship between connectivity parameters and the clinical UPDRS scores by performing correlation analyses between connectivity parameters during DBS-OFF and clinical score during OFF, connectivity parameters during DBS-ON and clinical score during ON, as well as percentage change in connectivity parameters and percentage change in clinical score.

## Results

### Clinical response and motor task data

Clinical responses as measured by the UPDRS-III scores are shown in *Table 1*. The mean improvement was 27.5 points (56.7% improvement, p<10^−6^). Similar improvements were also seen when the task data was analysed. Left hand movement durations and reaction times were decreased in the ON condition by an average of 28.87% (p = 0.002), and 20.33% (p = 0.025) respectively. The mean movement duration during ON and OFF were 0.82 s and 1.27 s respectively. The mean reaction times during ON and OFF were 0.63 s and 0.83 s respectively. Post-operative MRI – employing fine cuts through the STN – confirmed that each electrode contact lay within or overlapped the anatomical border of the STN in both axial and coronal views.

Scanning proceeded with no adverse events or change in post-scan UPDRS-III scores. Re-introduction of PD medications led to restoration of baseline motor function. Post-scan inspection of the IPG revealed DBS stimulation parameters and circuit impedance were unchanged.

### Hardware-related Artefact

**Figure 2:**
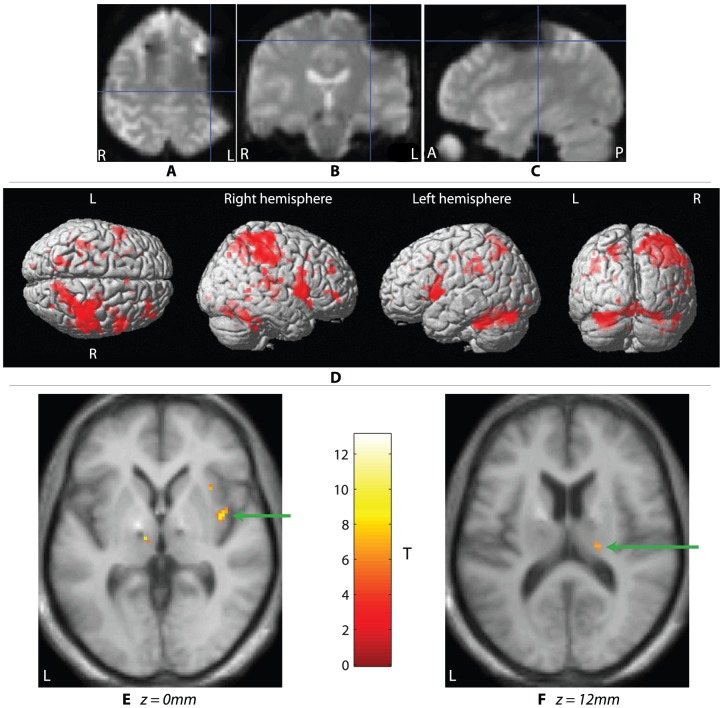
Imaging results. A typical drop-out artefact in a single subject's GE-EPI acquisition viewed from (a) axial, (b) coronal, and (c) sagittal sections; cross-hair position = −34.8, −21.5, 53.3 mm (MNI coordinates). SPMs in (d) summarize the movement network on a rendered MNI brain (p<0.001 uncorrected). Clusters representing BOLD signal increases in the insula cortex (e, *green arrow*), and thalamus (f, *green arrow*).

All GE-EPI scans suffered dropout artefact thought to be caused by the subgaleal connectors between the leads and extension cables sited over the left parietal bone (see *Figure 2a–c*).

#### Regional interactions between movement and stimulation

The main effect of movement was in accordance with previously published accounts (Limousin et al., 1997). The purpose of this contrast was to establish a network of functionally specialised nodes associated with task performance in our cohort. A summary rendered SPM of the network is displayed in *Figure 2d*.

Adjustment for clinical response to stimulation did not affect the regions that demonstrated highest levels of peak level BOLD response, although it did increase the significance levels of most of the areas described.

Our initial restricted volume analysis of the interaction (movement×stimulation) revealed robust increases in BOLD responses in the right insula cortex, and right thalamus (p<0.05, FWE corrected) when DBS was active. *See* *Figure 2d,e* *.*

Subsequent whole brain analysis of the interaction (movement×stimulation), using uncorrected thresholds (p<0.0005) revealed additional increases in the left superior frontal gyrus (Premotor area, PM, BA 6) and middle frontal gyrus (BA 10/46, DLPFC), right intra-parietal sulcus (hIP1), and inferior frontal gyrus pars triangularis (BA 45). See *Table 2*.

**Table 2:** The interaction between movement and stimulation identified using a whole-brain analysis.

Cluster Size/Voxels	T	Z	p(unc)	Small Volume Corrected p(FWE corrected)	MNI Coordinates (mm)			Anatomical Location		Functional/Brodmann Area
					x	y	z			
20	13.08	4.64	1.78E-06	-	−24	−2	62	Left	Superior Frontal Gyrus	Area 6
6	10.14	4.27	9.78E-06	-	−40	42	20	Left	Middle Frontal Gyrus	Area 47
**49**	**9.19**	**4.12**	**1.86E-05**	**0.005**	**38**	**−2**	**0**	**Right**	**Insula cortex**	
19	8.93	4.08	2.24E-05	-	38	30	16	Right	Inferior Frontal Gyrus (p. Triangularis)	Area 45
6	8.91	4.08	2.28E-05	-	−24	−14	16	Left	Putamen	
**7**	**8.61**	**4.03**	**2.84E-05**	**0.017**	**18**	**−24**	**10**	**Right**	**Thalamus**	
12	8.38	3.98	3.39E-05	-	20	−36	56	Right	Postcentral Gyrus	Area 3b
23	8.34	3.98	3.50E-05	-	38	−40	34	Right	Anterior Intra-parietal Sulcus	hIP1
5	8.24	3.96	3.77E-05	-	−10	−16	0	Left	Thalamus	
7	8.04	3.92	4.40E-05	-	−30	−22	40	Left	Postcentral Gyrus	Area 3a
9	7.59	3.83	6.36E-05	-	32	14	4	Right	Insula cortex	

BOLD activity increases comparing STN DBS ON v OFF during left hand movements (p<0.0005, uncorrected, Cluster size>5 voxels). Regions in **bold** were identified in the restricted volume analysis to the motor network described in the text (p<0.05, FWE corrected). The remaining regions are reported in view of strong support of their involvement from previous publications.

### Dynamic Causal Modelling: Bayesian Model Selection

**Figure 3:**
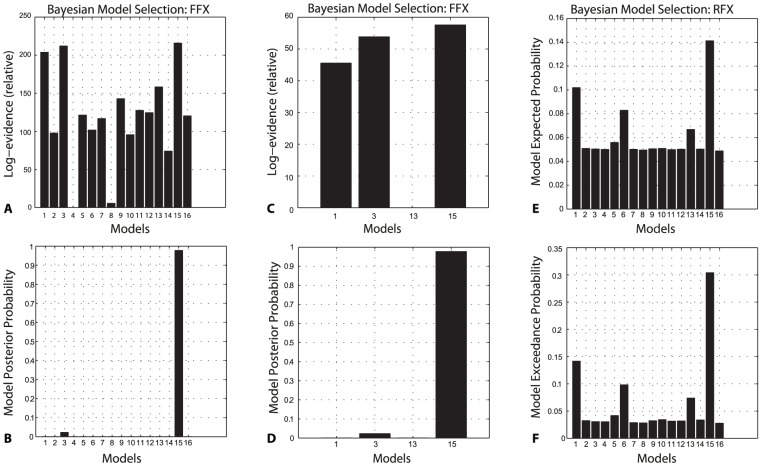
BMS results. FFX = Fixed Effects Assumptions, RFX = Random Effects Assumptions. (a) The relative log-evidences across all 16 specified models with model 3 showing the highest log-evidence. (b) Given the observed data and the models specified, one can be >95% certain that model 15 is the data generator. (c, d) The relative log-evidences between the 4 most likely models – again highlighting model 15 as the most likely model. This is repeated using RFX BMS, confirming the FFX findings (e, f).

In order to explore the effective connectivity between the insula cortex and thalamus, we constructed 16 models of connectivity and used Bayesian Model Selection to determine the most likely model to produce our data. The relative log-evidences across all models for all participants are shown in *Figure 3a,b*.

Across all models, model 15 emerges as the most likely, followed by 3 and 1. The difference in relative log-evidences, ΔF, are 3.77 and 8.25 respectively, indicating that there is very strong evidence in favour of model 15 [37]. Restricting the BMS to the four most likely models illustrates this more clearly (*3e,f*). Relative F values for each model – for each subject – confirmed models 15, 3 and 1 consistently scored highly. Random effects BMS produced similar results, confirming model 15 as the most likely of all the models to be the generator of the data (*3g,h*).

### Dynamic Causal Modelling: Parameter Estimates

Effective connectivity estimates (corresponding to the DCM A-matrix) reveal that during voluntary movements activity in the insula cortex had an inhibitory (cortico-thalamic) drive on the thalamus, whereas thalamic activity had an excitatory (thalamo-cortical) effect on the insula cortex. Note, in DCM, regions are often used to ‘stand in’ for multiple systems, and effective connectivity is polysynaptic. In our context, the insula region is taken as representative of a cortical system, while the thalamus summarises subcortical responses. The thalamo-cortical-cortico-thalamic loops most probably comprise several synaptic relays.

Crucially, DBS effectively reversed this extrinsic coupling, changing cortico-thalamic connectivity from inhibitory to excitatory, whereas the thalamus assumed an inhibitory effect on the activity in the insula cortex under DBS. In other words, DBS appears to sensitise subcortical responses to cortical afferents, with a reciprocal desensitisation of cortical responses to subcortical projections. This is a remarkable reversal of effective connectivity that is the quantitatively largest (to our knowledge) that has been reported in the DCM literature. There was no significant correlation between cortico-thalamic connectivity parameters and the clinical scores (P<0.05) to suggest a direct linear relationship between these indices.

## Discussion

We have demonstrated that fMRI can be used to study the effects of therapeutic STN DBS on brain activity without compromising patient safety or DBS function. All sequences used were informed by our previous on-site experiments, and we stress the importance of performing *on-site* assessments given the variability in scanner configurations [22], [23].

We specifically selected patients who were at least 6 months post-implantation of both their electrodes and IPGs, and exhibited a good response to DBS. This allowed us to study the effect of *chronic therapeutic* stimulation, unlike in former studies. Given DBS improves motor control in this cohort, we were also specifically interested in changes to the motor network; thus we chose to explore interactions principally within a network of nodes that we had confirmed were engaged by task performance.

Previous results have varied with the task employed; specifically whether it involves self-generated and/or externally-cued movements [10]–[12], [14], [36], [38]. The position of the electrodes within motor, limbic or associative STN sub-regions may also contribute to the variability of previous reports.

We first confirmed the reliability of fMRI in these patients, defining regions specialized for task performance that were in accordance with the literature [36]. When testing for the movement×stimulation interaction within those nodes, we found that DBS-associated response increases were most prominent within the insula cortex and the thalamus.

The insula cortex appears to be functionally heterogeneous, displaying two independent patterns of *functional* connectivity; anterior cortex activity *correlates* with frontal/cingulate regions mediating attention or salience, and the posterior cortex possesses dense sensorimotor connectivity [39], [40]. Previous reports demonstrate that PD patients show reduced insula cortex activation during self-generated movements [41], which are known to be impaired in these patients. Tractography has identified pathways connecting the posterior insula cortex to the motor STN, and anterior insula cortex to the limbic STN [42], consistent with reports of posterior insula lesions resulting in hemiballismus [43]. DBS has been found to modulate activity here previously in a single case study; however, lower significance thresholds were used [15]. Taken together with our findings, there is now more robust evidence to implicate this region in motor processing and the successful performance of self-generated movements.

However, therapeutic effect may not just be related to augmentation of specialized motoric cortex. The insula cortex has previously been implicated in coupling of auditory stimuli and motor outputs [44]. DBS may also affect limbic circuitry, known to involve the STN. However, our lack of any anterior cingulate cortex modulation suggests that STN DBS in our cohort is not primarily modulating limbic networks during performance of our motor task. This may be related to our routine targeting of the postero-lateral (motor) STN.

Previous studies have suffered from limited spatial resolution making subcortical structures difficult to examine; however, thalamic modulation has also been previously identified in pilot fMRI studies [45], [46]. Given that the orthodromic output of the STN ultimately projects to the thalamus, our finding of increased thalamic BOLD response associated with STN DBS is in-keeping with electrophysiologically established orthodromic effects of STN stimulation on the thalamus [47]–[49].

Additional interactions were found in the intra-parietal sulcus, IFG, PM and DLFPC (p<0.0005, uncorrected). While these changes did not survive FWE correction, their detection at stringent uncorrected thresholds, high z-scores (max = 4.64), and their accordance with previous literature [10], [36], merit inclusion in this report.

However, as discussed, the architecture underlying neural processing relies on two fundamental principles; functional specialization and functional integration [50]. Our conventional SPM analysis fails to convey how the effective connectivity between the modulated regions is affected by DBS. This interaction has thus far been overlooked in the literature.

Due to the small volume of the STN itself, and electrode artefact masking it, we were unable to measure activity in STN proper and its relationship with the cortex. The models we explored permitted us to establish which regions showed a non-specific (intrinsic) DBS–induced change in gain or sensitivity, and which inputs showed a specific increase of gain to particular (extrinsic) afferents. DCM does not distinguish between monosynaptic or polysynaptic connections; therefore we are not suggesting direct insula-thalamic connectivity, rather, activity most likely flows via the BG nuclei. Similarly, the intrinsic effective connectivity ‘within’ the thalamus is likely to include loops that pass through the BG.

Our primary finding is that therapeutic DBS alters cortico-thalamic coupling. Our winning model stipulates that in PD patients with DBS switched OFF, the insula has an inhibitory influence on the thalamus during movement. However, therapeutic stimulation was found to reverse this by sensitizing the subcortical systems to its afferents. In other words, changes in thalamic response appear to be related to both cortical and BG afferents. Furthermore, DBS reversed the cortical response to thalamic projections, overall having an inhibitory effect.

The changes in effective connectivity were associated with an improvement in task performance and clinical measure of PD disability, potentially suggesting that these changes in fact facilitate harmonious integration within cortico-thalamo-cortico loops. This is a novel finding and is distinct from attempts to explain DBS's therapeutic effects in terms of regional changes in neural activity or sensitivity alone. DCM has previously been shown to be robust and sensitive to detecting changes in cortical motor network coupling between PD patients and controls, as well as before and after dopaminergic medication [30]. We have demonstrated here that DCM can also be sensitive to the modulatory actions of DBS. The lack of significant correlation between cortico-thalamic connectivity parameters and the clinical score indicates that there may be a more complex non-linear relationship. Clearly, the current (two region) DCMs are an over-simplification and we anticipate a more comprehensive modelling of distributed cortical and subcortical responses in future work.

**Figure 4:**
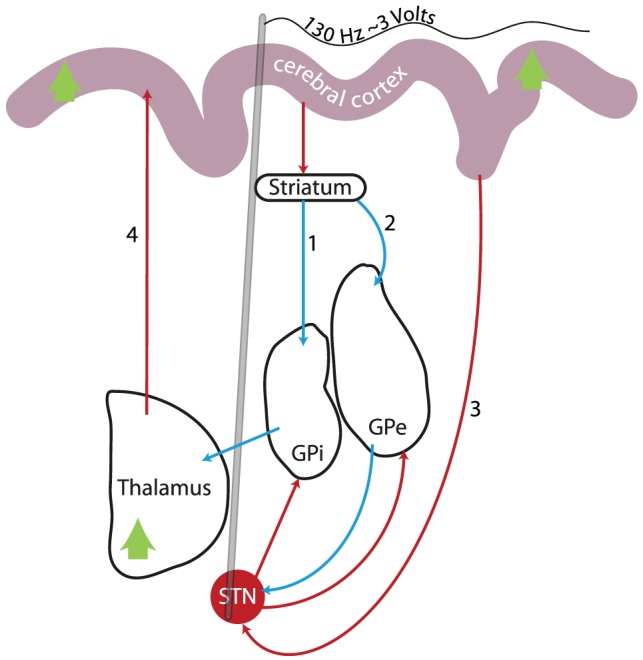
Simplified diagram of current understanding of cortico-striato-pallido-thalamo-cortical circuitry. Green arrows represent the regions in which there were BOLD response increases. The 3 input pathways are shown; the direct (1), indirect (2), and hyperdirect (3) pathways. Thalamic ‘cortical afferents’ are likely to arrive via one of these pathways – passing through BG nuclei. The thalamic ‘BG afferents’ – discussed in the main text – arrive from other BG nuclei, independent of cortical activity. Red arrows indicate glutamatergic (excitatory) projections; blue arrows indicate GABAergic (inhibitory) projections. The grey line represents the DBS electrode. GPe/GPi = Globus Pallidus pars externa/interna. STN = Subthalamic Nucleus.

The reversal of the DCM parameter estimates, representing a switch from predominantly inhibitory cortico-thalamic drive, to predominantly excitatory cortico-thalamic requires scrutiny. Traditional rate-based models of basal ganglia function [51], [52] suggest that the thalamus receives cortical inputs via the nuclei of the basal ganglia (*Figure 4*). Thalamic response to cortical excitation depends on the pathway through which the signal is propagated; the hyperdirect and indirect pathways cause an excitation of the output nuclei (GPi/SNr), resulting in thalamic inhibition. Transmission via the direct pathway however inhibits the output nuclei, disinhibiting thalamic neurons.

The documented shift from inhibition to excitation of the thalamus may suggest that DBS shifts the sensitivity of the thalamus; from pathways that result in an inhibitory effect on thalamic neurons, e.g. hyperdirect or indirect pathway afferents, to pathways which ultimately result in thalamic neuronal excitation, e.g. afferents from the direct pathway.

More contemporary understanding however suggests that firing pattern may be more relevant to motor deficits in PD than the firing rate, and DBS re-sets the pallidum into a regular, ordered pattern, overcoming the ‘pathological’ PD pattern, a process that has been referred to as *‘jamming’* [53], [54]. Combined computational and electrophysiological studies have found that the response of thalamic neurons to excitatory cortical inputs is down-regulated by the presence of disordered pallidal inputs, yet is restored by therapeutic STN DBS [53], [55], [56]. Pallidal modulation of thalamic response could similarly explain the reversal of cortico-thalamic coupling we have documented here.

Given the ‘hyperdirect’ connectivity between the STN and insula cortex, DBS may similarly alter cortical afferents to the STN, causing a change in the behaviour of cortical neurons. This supports recent claims from the animal literature that antidromic stimulation of axons projecting to the STN produces complex activations of cortical circuits [57], which might also be responsible for the clinical effect of DBS [5]. Further studies employing electrophysiological techniques may be required to provide deeper insights into the synaptic mechanisms involved.

### Limitations of this study

The extension cables sited over left parietal bone created a drop-out susceptibility artefact on GE-EPI acquisitions, partially obscuring left sensorimotor regions. Given our careful preprocessing, including the use of field map correction and a bespoke normalisation procedure, there is no evidence to suggest that BOLD signal from the remaining brain regions were affected by this artefact, especially given the ‘main effect of movement’ contrast produced a network of well-described motor regions. Previous studies involving implanted electrodes have confirmed that similar artefacts do not significantly impair the functional data [58].

Only patients who had a significant therapeutic response to stimulation (minimum UPDRS-III improvement in our sample was 38.5%) were included in our study. While this permitted novel investigation of the modulatory effects of *confirmed therapeutic* stimulation, our conclusions should only be applied to patients who have shown such improvements.

We included a covariate for hand dominance to minimize variability vis-à-vis laterality of function. Other studies investigating motor control generally use right hand movements, complicating comparisons with other studies, but this was unavoidable given our standard surgical practice of placing the connector to electrode extension cables subcutaneously over the left parietal bone.

The T/R head coil was used to minimize RF exposure to the DBS instrumentation circuit *in situ*. Such coils are not regularly used in fMRI studies as they forgo the signal to noise ratio advantages of conventional multi-channel receive-only head coils. This may explain why regions previously reported in the literature and identified in our whole-brain search at uncorrected thresholds (including the premotor cortex, and DLPFC) did not survive FWE correction. While this was unavoidable given the safety concerns, we nevertheless identified two regions that are irrefutably associated with therapeutic STN stimulation, which formed the subsequent focus of the more sophisticated network modelling possible with fMRI data.

Our results cast the therapeutic effects of DBS in a fundamentally new light, emphasising a role in changing distributed cortico-subcortical interactions in a way that has not been previously explored. Investigating the effective connectivity changes induced by DBS *in vivo* represents a new avenue of study that may shed light on its underlying mechanisms of action. Given our modest sample size and small network, we stress that further work is required to verify and validate our findings. Selection of the target nucleus for DBS is pivotal to producing the desired therapeutic effect. Historically this has relied upon stimulating nuclei that had previously been targeted for ablative procedures. Understanding the impact that stimulation is having on the networks that course through the target may allow for improvements in current targeting, as well as rational selection of novel targets to extend its use to patients with other disabling conditions.
